# The relation between neutrophil-to-lymphocyte ratio and coronary chronic total occlusions

**DOI:** 10.1186/1471-2261-14-130

**Published:** 2014-09-27

**Authors:** Kenan Demir, Ahmet Avci, Bulent Behlul Altunkeser, Ahmet Yilmaz, Fikret Keles, Ahmet Ersecgin

**Affiliations:** Faculty of Medicine Cardiology Department, Selcuk University, 42075 Konya, Turkey

**Keywords:** Chronic total occlusions, Neutrophil-to-lymphocyte ratio, SYNTAX score

## Abstract

**Background:**

Neutrophil-to-lymphocyte ratio (NLR) is a marker of systemic inflammation that correlates with cardiac events. This study assessed the association between NLR and the presence of chronic coronary total occlusion (CTO).

**Methods:**

The study population included 225 patients, a control group (n = 75), a coronary artery disease group (n = 75), and a CTO group (n = 75). NLR was compared in the three groups.

**Results:**

NLR levels were significantly higher in the CTO than in the other two groups (p < 0.001). Bivariate correlation analysis showed a positive correlation between NLR and SYNTAX Score, and multivariate logistic regression analysis found that NLR was an independent predictor of CTO. ROC analysis showed that an NLR cut-off of 2.09 could distinguish between patients with and without CTO (AUC = 0.74; 95% CI, 0.68–0.81), with a specificity of 69.3% and a sensitivity of 61%.

**Conclusion:**

NLR may be useful as a marker of CTO.

## Background

Cardiovascular disease is the major cause of death in developed countries. Atherosclerosis is a progressive disease characterized by the accumulation of lipids and fibrous elements in the large arteries and constitutes the single most important contributor to the growing burden of cardiovascular disease [1]. Inflammation plays a key role in the development, progression, and complications of atherosclerosis. Inflammatory processes interacting with endothelial dysfunction initiate a progressive process within the arterial wall, resulting in the reduction or obstruction of blood supply to end organs of the body including the brain, heart, and intra-abdominal organs, as well as tissues of the lower limbs, causing morbidity and mortality [2–4]. Increased cardiovascular risk has been found to correlate with counts of white blood cells (WBC) and subtypes. Neutrophil-to-lymphocyte ratio (NLR) is a marker of systemic inflammation that has been found to correlate with mortality and cardiac events in many cardiovascular diseases such as stable coronary artery disease (CAD), unstable CAD, and acute decompensated heart failure [5, 6].

Chronic coronary total occlusions (CTOs) are generally considered lesions of duration >3 months in which the vessel shows either complete interruption of antegrade blood flow on angiography or minimal contrast penetration through the lesion without distal vessel opacification [7]. CTO, which is present in one third of patients with coronary diseases, is the end stage of coronary artery atherosclerosis [8].

The SYNTAX score (SS) provides important information on favorable revascularization strategy and the prognostic significance of CAD [9]. NLR may be associated with a greater complexity of CAD and CTO as assessed using the SS. Thus, this study was designed to examine whether NLR is associated with the extent of CAD and CTO.

### Patients and methods

The study population included 225 patients who were referred for elective coronary angiography for stable angina pectoris between August 2013 and April 2014. All patients recruited in this study underwent coronary angiography for the presence of chest pain with objective signs of ischemia (treadmill exercise test or myocardial perfusion scintigraphy). Exclusion criteria were previous coronary artery bypass grafting (CABG), acute coronary syndrome, hematological disease, malignancy, severe renal or liver disease, ongoing infection or chronic inflammatory disease, and autoimmune disease. Patients (n = 75) who were considered to have lesions with a duration of >3 months, in which the vessel shows no antegrade blood flow on angiography or only minimal contrast penetration through the lesion without distal vessel opacification were included in the CTO group. Patients (n = 75) who had coronary lesion with a diameter stenosis of ≥50% were included in the CAD group, and those (n = 75) who had normal coronary artery or coronary lesion with a diameter <50% were included in the control group. Patients’ laboratory and clinical characteristics, such as age, sex, diabetes mellitus, hypertension, hypercholesterolemia, smoking, and family history of cardiovascular disease were reported. Hemoglobin, WBC, platelet, lymphocyte, and neutrophil counts were measured as part of the automated complete blood count. Baseline NLR was measured by dividing the neutrophil count by the lymphocyte count.

Transthoracic echocardiographic examination was conducted in all patients with Vivid E9 system using a 1.5-4.6 MHz probe (GE-Vingmed Ultrasound AS, Horten, Norway) before they were discharged. The left ventricular ejection fraction was measured using the modified Simpson rule [10].

SS is an angiographic tool used in grading the complexity of CAD. Each coronary lesion with a diameter stenosis more than 50% in vessels >1.5 mm must be scored. The online latest updated version (2.1) was used in the calculation of the SS (http://www.syntaxscore.com) [11].

The study was approved by the ethical committee of Selcuk University, Faculty of Medicine. All participants also had to give a written consent.

### Statistical analysis

SPSS 17.0 for Windows was used for statistical analyses (SPSS, Chicago, Illinois, USA). Continuous variables were presented as median or mean ± SD; categorical variables were defined as percentage. Differences in the continuous variables between groups were determined using Student’s t test or the Mann-Whitney U test for variables with or without normal distribution, respectively. To test the normal distribution, the Kolmogorov-Smirnov test was used. Categorical variables were summarized as percentages and compared with the *X*^2^ test. The Pearson correlation coefficient was computed to examine the association between 2 continuous variables. Logistic regression analysis with “enter” method was performed including independent variables being significantly different between patients with CAD and CTO and having a possible causative role for CTO. All tests of significance were two tailed. An exploratory evaluation of additional cut-points was performed using the receiver operating characteristic (ROC) curve analysis. Statistical significance was defined as a p value of less than 0.05.

## Results

**Figure 1:**
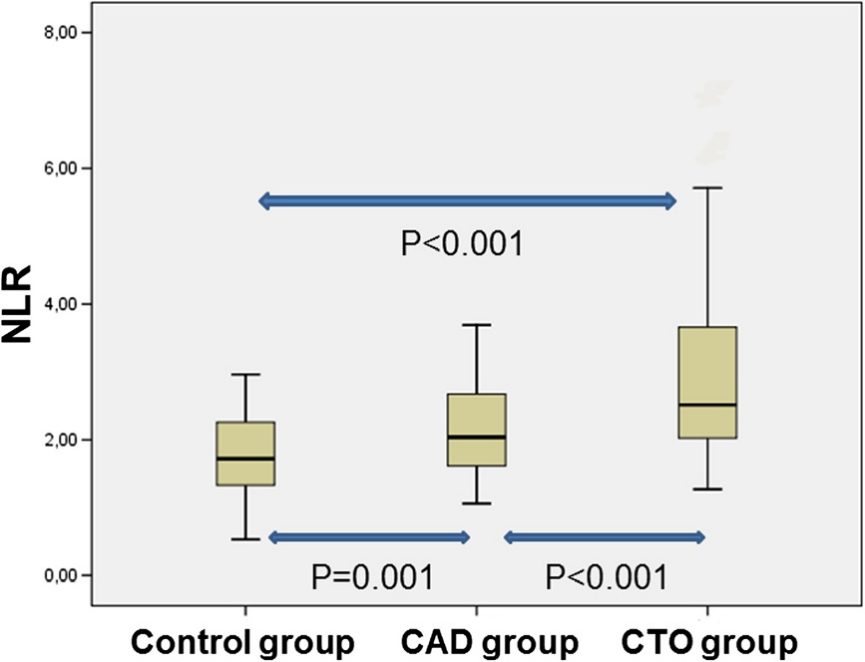
**NLR levels among study groups.**

The baseline characteristics of patients are summarized in Table 1. There were 75 patients (mean age of 58 ± 11, and 45.3% were male) in the control group, 75 patients (mean age of 63 ± 10, and 65.3% were male) in the CAD group, and 75 patients (mean age of 61 ± 8, and 88% were male) in the CTO group. Mean age was significantly higher in the CAD group (p = 0.01). The male patient ratio of CTO and CAD groups were higher than that of the control group. The prevalence of cardiovascular risk factors. such as diabetes mellitus, hyperlipidemia, and family history of CAD, were significantly higher in the CTO group than in other groups (p = 0.004, p < 0.001, and p < 0.001, respectively), but hypertension and smoking were similar in all the groups. The groups were similar in terms of hemoglobin levels and platelet count. WBC and neutrophil count were significantly higher in the CTO group; in contrast, the lymphocyte count was significantly lower in the CTO group (p = 0.001, p < 0.001, and p = 0.01, respectively). NLR levels were significantly higher in the CTO group (p < 0.001) (Figure 1). Also, Gensini score and SYNTAX score were significantly higher in the CTO group than in other groups (both p < 0.001).

**Figure 2:**
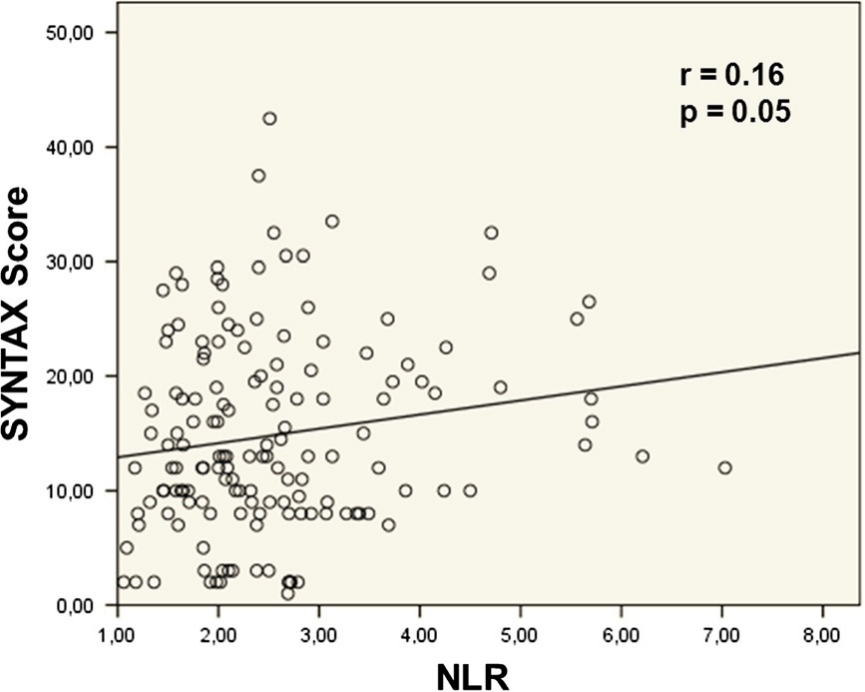
**Correlation between NLR and SYNTAX score; Pearson test was used.**

**Figure 3:**
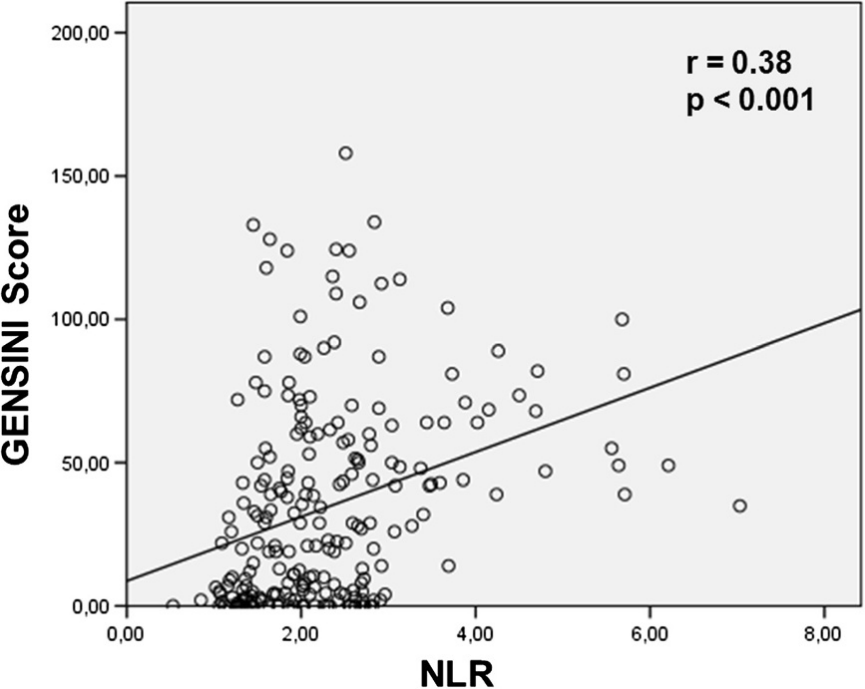
**Correlation between NLR and Gensini score; Pearson test was used.**

In the bivariate correlation analyses, there was a weak positive correlation between NLR and SS (p = 0.05) (Figure 2). And, in the bivariate correlation analyses, there was a positive correlation between NLR and Gensini score (p < 0.001) (Figure 3).

**Table 1:** **Baseline clinical characteristic of patients and controls**

	Control group (n = 75)	CAD group (n = 75)	CTO group (n = 75)	p
Age, years	58 ± 11	63 ± 10	61 ± 8	**0.01**
Male, n (%)	34 (45.3)	49 (65.3)	66 (88)	**<0.001**
Diabetes mellitus, n (%)	12 (16)	30 (40)	26 (34.7)	**0.004**
Hypertension, n(%)	50 (66.7)	55 (73.3)	49 (65.3)	0.52
Hyperlipidemia, n (%)	19 (25.3)	44 (58.7)	52 (69.3)	**<0.001**
Family history of CAD, n (%)	2 (2.7)	4 (5.3)	29 (38.7)	**<0.001**
Smoking, n (%)	25 (33.3)	33 (44.4)	39 (52)	0.06
Ejection fraction, %	57.9 ± 7.4	55.1 ± 10	51.3 ± 9.1	**<0.001**
Hemoglobin, g/dL	14.2 ± 1.6	13.8 ± 1.6	13.7 ± 1.7	0.16
Platelet, 10^3^/ml	233 ± 61	251 ± 75	233 ± 68	0.17
WBC, 10^3^/ml	7.40 ± 1.84	7.93 ± 1.96	8.63 ± 1.94	**0.001**
Neutrophil, 10^3^/ml	4.16 ± 1.27	4.78 ± 1.36	5.62 ± 1.49	**<0.001**
Lymphocyte,10^3^/ml	2.46 ± 0.76	2.33 ± 0.67	2.11 ± 0.72	**0.01**
NLR	1.79 ± 0.57	2.13 ± 0.64	2.95 ± 1.29	**<0.001**
Gensini score	1.5 (0-13)	26 (2-128)	64 (33.5-158)	**<0.001**
SYNTAX score		10.3 ± 6.8	19.2 ± 7.6	**<0.001**

CAD, coronary artery disease; CTO, coronary total occlusion; NLR, neutrophil-to-lymphocyte ratio; WBC, white blood cell.

Significant p values are bold.

In the multivariate logistic regression analysis, NLR, male sex, hyperlipidemia, and family of history CAD were identified as independent predictors of CTO in our model (Table 2).

**Figure 4:**
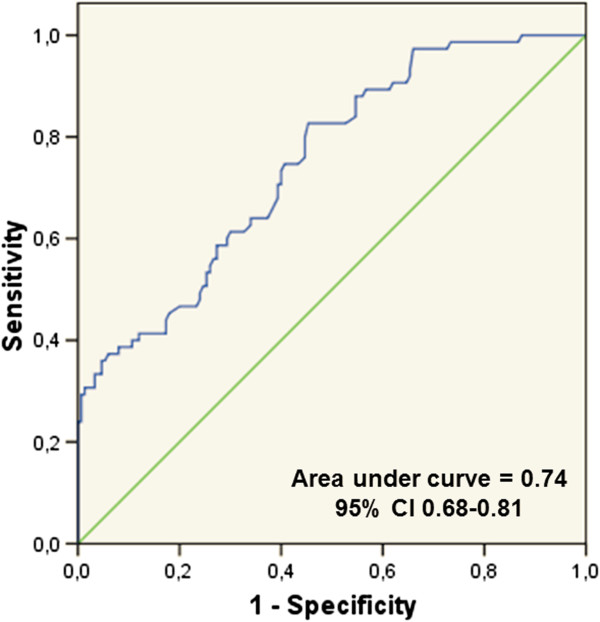
**Receiver–operating characteristic analysis and curve for predicting coronary total occlusions (CTOs).**

In ROC analysis, a cut point of 2.09 for NLR was identified in patients with CTO (area under the curve = 0.74; 95% CI, 0.68-0.81). A NLR value of more than 2.09 demonstrated a specificity of 69.3% and a sensitivity of 61% (Figure 4).

**Table 2:** **Predictors of CTO in multivariate logistic regression analysis**

Independent variables	β ± SE	Wald	p
NLR	1.03 ± 0.25	16.74	**<0.001**
WBC	0.18 ± 0.09	3.79	0.05
Sex	-1.36 ± 0.45	9.11	**0.003**
Hyperlipidemia	-0.81 ± 0.38	4.56	**0.03**
Family history of CAD	-2.45 ± 0.55	19.65	**<0.001**

β ± SE, beta ± Standard error; CAD, Coronary artery disease; NLR, neutrophil-to-lymphoycte ratio; WBC, white blood cell.

Significant p values are bold.

## Discussion and conclusions

To our knowledge, this study is the first to investigate the relationship between NLR and CTO. This study found that NLR levels were significantly higher in the CTO group, suggesting that high NLR levels may predict CTO in patients with CAD.

Atherosclerosis is a complex and multifactorial inflammatory disease characterized by low-grade arterial inflammatory lesions that can develop through disease progression [12]. Previous epidemiological and clinical studies have shown a clear association between peripheral leukocyte counts and the risk of adverse cardiovascular events in patients with CAD [6]. WBC subtypes have also been shown to be markers of inflammation in cardiovascular diseases [13]. NLR is a predictor of severe atherosclerosis that may be useful for cardiac risk stratification in patients with CAD [14]. Several studies have suggested that high NLR levels were associated with adverse outcomes and increased cardiovascular mortality in patients with stable CAD and acute coronary syndrome and in those undergoing CABG and primary percutaneous coronary intervention [15–18].

The SS is an established anatomical-based tool for objectively determining the complexity of coronary artery disease and for guiding the decision on whether to perform CABG surgery or percutaneous coronary intervention. The SS assesses the importance of a diseased coronary artery segment based on its severity, anatomical location, and importance in supplying blood to the myocardium; the SS can also be used to assess bifurcation lesions, and total occlusion characteristics [19]. The Gensini score is another scoring system used to evaluate collateral circulation in the coronary arteries [20]. CTO is defined as complete occlusion of the coronary vessel with TIMI 0 flow, present for an estimated duration of ≥3 months [21].

Elevated inflammatory markers and WBC counts have been reported associated with the extent and severity of CAD [22]. Additionally, WBC and neutrophil counts were found to be higher in patients with high than low SS [23, 24]. High SS has been found to be predict poorer short and long-term clinical outcomes in patients with CAD who underwent revascularization [25]. Increased NLR, widely recognized as an indicator of systemic inflammation, has been associated with higher mortality rates (Papa et al) [15]. Moreover, NLR has been found to correlate with the severity and complexity of CAD, assessed by SS in stable patients with CAD (Kaya et al.) [26], and higher NLR at baseline was found to be independently associated with greater coronary complexity of CAD as assessed by SS (Sönmez et al.) [9]. In this study, both Gensini score and SS were significantly higher in the CTO than in the CAD group, with bivariate correlation analysis showing a weak positive correlation between NLR and SS.

Arterial CTOs in the coronary and peripheral vasculature are common and are associated with significant morbidity and adverse outcomes [27]. CTO develops from total luminal obstruction of an artery by a thrombus, with subsequent organization and varying degrees of recanalization; often, these events are clinically silent. The process of thrombus organization coincides with the development of intraluminal microvessels accompanied by inflammatory cells, followed by infiltrating smooth muscle cells and deposition of proteoglycan matrix [28]. In CTOs of all ages, a close relation in both location and intensity has been observed between cellular inflammation and vessel wall neovascularization [29]. NLR is an indicator of inflammatory status that can easily be derived from the WBC count. Moreover, higher NLR has been correlated with the development of poor coronary collateral circulation in patients with CTO (Kalkan et al.) [30]. To our knowledge, our study is the first to evaluate NLR levels in controls, patients with CAD, and patients with CTO, finding that NLR levels were significantly higher in the CTO than in the other groups. An optimal NLR cut off value of 2.09 was found to predict CTO with a specificity of 69.3% and a sensitivity of 61%. Moreover, multivariate logistic regression analysis showed that NLR was an independent predictor of CTO. These findings indicate that measurements of NLR, a novel cardiovascular risk marker, is an important, simple, and inexpensive method of screening for CTO prior to the performance of other expensive and invasive procedures.

The major limitations of our study include the small patient population and the lack of measured inflammatory markers. Moreover, our exclusion criteria prevent the extrapolation of our findings to other populations with these co-morbid conditions.

In conclusion, NLR may predict the presence of CTO, but additional studies with more patients are required to confirm this result.
